# Supplementary material for: 15-cis-Phytoene Desaturase and 15-cis-Phytoene Synthase Can Catalyze the Synthesis of β-Carotene and Influence the Color of Apricot Pulp
Source: Foods. 2024 Jan 17;13(2):300. doi: 10.3390/foods13020300 (PMC10815377; doi:10.3390/foods13020300)
Supplement: Supplementary file 1 [file foods-13-00300-s001.zip › foods-2798747-supplementary.pdf]

Supplementary Data

For

15-*cis*-Phytoene Desaturase and 15-*cis*-Phytoene Synthase Can Catalyze the Synthesis of β-Carotene and Influence the Color of Apricot Pulp

Ningning Gou [1], Xuchun Zhu [2], Mingyu Yin [1,3], Han Zhao [1], Haikun Bai [1], Nan Jiang [1], Wanyu Xu [1], Chu Wang [1], Yujing Zhang [1] and Tana Wuyun [1,*]

[1] Kernel-Apricot Engineering and Technology Research Center of State Forestry and Grassland Administration, Research Institute of Non-Timber Forestry, Chinese Academy of Forestry, State Key Laboratory of Tree Genetics and Breeding, Zhengzhou 450003, China
[2] School of Food and Health, Beijing Technology and Business University, Beijing 100037, China
[3] Institute of Ecological Conservation and Restoration, Chinese Academy of Forestry, Key Laboratory of Desert Ecosystem and Global Change, National Forestry and Grassland Administration, Beijing 100091, China
[*] Correspondence: wuyuntana@caf.ac.cn

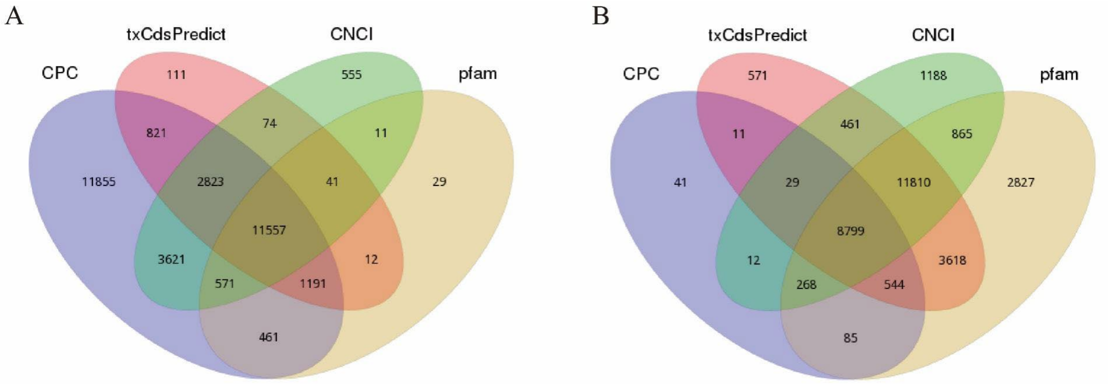

Supplementary Figure S1. The Venn diagram shows the transcriptome coding ability predicted by CPC, txcdspredict, CNCI, and Pfam databases. **A.** predicted result Venn Diagram-lncRNA. **B.** predicted result Venn Diagram-lncRNA.

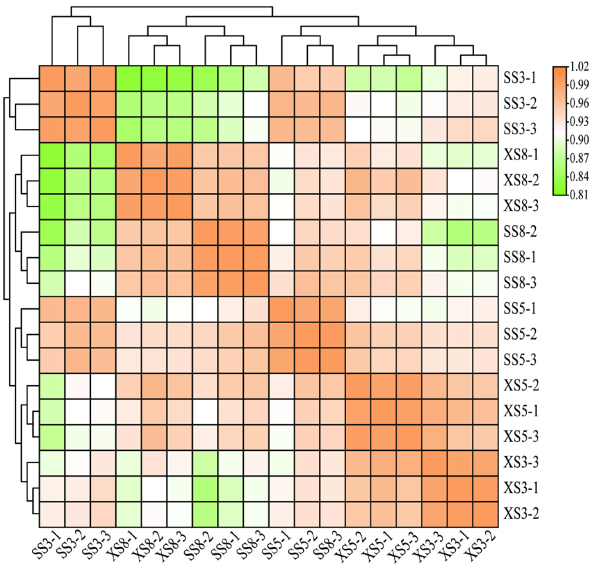

Supplementary Figure S2. Correlation clustering heat maps of different comparison groups.

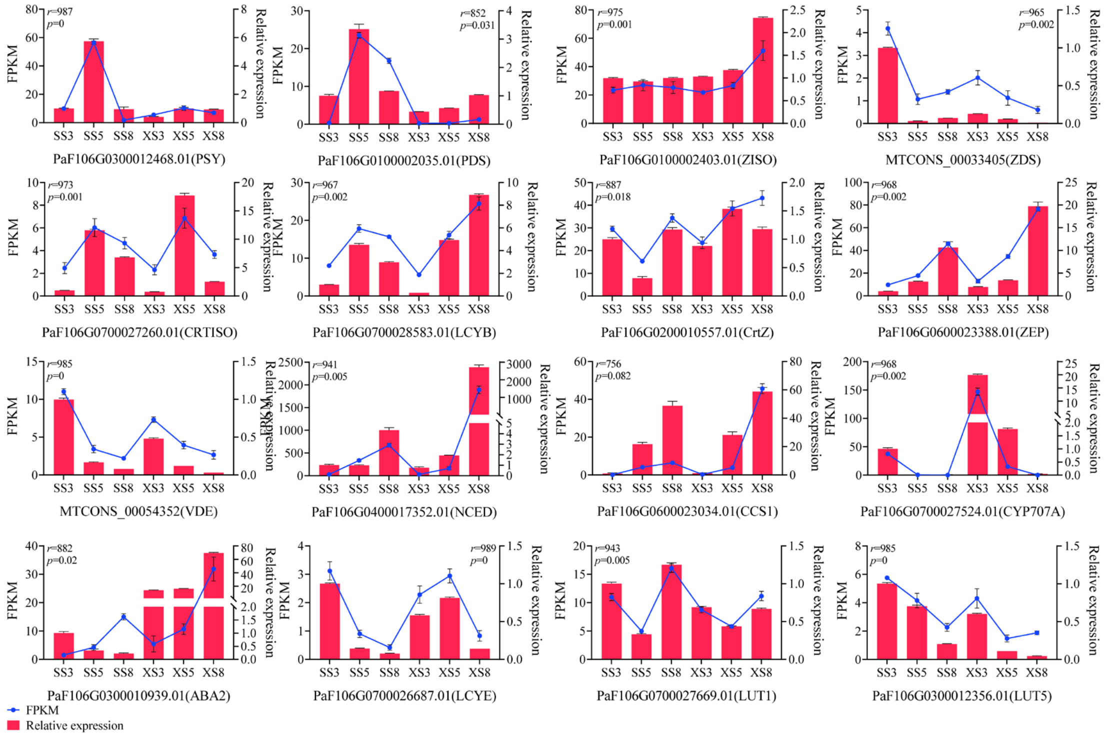

Supplementary Figure S3. Expression analysis of 16 differentially expressed mRNAs by qRT-PCR in orange and white apricot during S3, S5 and S8 stage. Columns and lines indicate qRT-PCR and RNA-seq of the candidate differentially expressed genes, respectively. The Pearson correlation coefficient is denoted by r, and sample significance is denoted by P.

Table S1. Experimental sample information.

Number	Cultivar	Pulp color	Sampling position	Sampling stage
OFP1	yuxing	Orange		mature stage
OFP2	shenyangdashuaifuxing	Orange		mature stage
OFP3	qingyangxing	Orange		mature stage

OFP4	ningxiandajiexing	Orange		mature stage
OFP5	JTY	Orange		9 developmental stages
OFP6	jinshahong	Orange		mature stage
OFP7	erhongbadanxing	Orange		mature stage
OFP8	dongxing	Orange		mature stage
OFP9	chuanzhihongyouxi	Orange		mature stage
OFP10	X12	Orange		mature stage
OFP11	31-2-6-11	Orange		mature stage
OFP12	11Z05	Orange		mature stage
OFP13	11N02	Orange		mature stage
OFP14	11D02	Orange		mature stage
OFP15	1-16-11-1	Orange		mature stage
OFP16	10-10-21-6	Orange	the experimental	mature stage
OFP17	20191008	Orange	field of the	mature stage
OFP18	20191007	Orange	Research Institute	mature stage
OFP19	20191006	Orange	of Non-timber	mature stage
OFP20	20190199	Orange	Forestry in	mature stage
OFP21	2018001	Orange	Yuanyang	mature stage
WFP1	zhenxing34	White	County, Henan	mature stage
WFP2	yuanyangbaixing	White	Province, China	mature stage
WFP3	qiuhong	White		mature stage
WFP4	luntai11hao	White		mature stage
WFP5	XX15	White		mature stage
WFP6	X15	White		9 developmental stages
WFP7	SS010	White		mature stage
WFP8	RV	White		mature stage
WFP9	PT54	White		mature stage
WFP10	PT15	White		mature stage
WFP11	LS-11	White		mature stage
WFP12	F70	White		mature stage
WFP13	F40	White		mature stage
WFP14	F31	White		mature stage
WFP15	77hangdi25ke	White		mature stage
WFP16	64hangdi2ke	White		mature stage
WFP17	38-ningxiandajiexing	White		mature stage
WFP18	mengzhoujiaxibaixing	White		mature stage
WFP19	11Z17	White		mature stage
WFP20	20191004	White		mature stage
WFP21	12099	White		mature stage
WFP22	12079	White		mature stage

Table S2. Whole transcriptome sequencing data filtering statistics.

Sample	TotalRawReads	TotalClean Reads	TotalCleanBase	GCofFq1	GCofFq2	Q20ofFq1	Q20ofFq2	Q30ofFq1	Q30ofFq2	CleanReads Ratio	TotalMappin gRatio	UniquelyMa ppingRatio	StrandSpecif icRatio
SS1-1	112988812	112004560	11200456000	47.33%	47.69%	98.06%	97.72%	93.91%	92.55%	99.13%	74.20%	71.17%	92.94%
SS1-2	127539558	126293984	12629398400	47.47%	47.79%	97.98%	98.05%	93.71%	93.57%	99.02%	74.57%	71.64%	95.03%
SS1-3	129383592	127986180	12798618000	47.73%	48.07%	98.03%	97.96%	93.84%	93.32%	98.92%	73.55%	70.43%	94.66%
SS2-1	129383592	128177864	12817786400	48.05%	48.36%	97.95%	97.98%	93.59%	93.28%	99.07%	78.09%	75.11%	96.34%
SS2-2	129383592	128225318	12822531800	48.18%	48.50%	97.93%	97.96%	93.61%	93.23%	99.11%	78.21%	75.24%	96.39%
S2-3	129383592	128024968	12802496800	47.82%	48.21%	97.96%	98.02%	93.72%	93.51%	98.95%	78.07%	75.19%	96.40%
SS3-1	129383592	128165018	12816501800	48.67%	48.87%	98.02%	97.91%	93.83%	93.16%	99.06%	79.77%	76.64%	95.93%
SS3-2	129383592	128126212	12812621200	48.62%	48.87%	98.07%	98.07%	94.01%	93.70%	99.03%	79.75%	76.70%	96.20%
SS3-3	129383592	128244312	12824431200	48.68%	48.95%	97.93%	98.10%	93.54%	93.71%	99.12%	79.79%	76.60%	95.94%
XS1-1	129383592	128132296	12813229600	47.52%	47.85%	97.99%	98.09%	93.71%	93.64%	99.03%	74.68%	71.63%	92.52%
XS1-2	127435434	126004654	12600465400	47.63%	47.99%	97.70%	96.91%	94.12%	92.33%	98.88%	73.35%	70.16%	92.83%
XS1-3	123623084	122030400	12203040000	48.03%	48.38%	97.87%	97.04%	94.57%	92.68%	98.71%	72.59%	69.27%	93.19%
XS2-1	121671572	120578976	12057897600	47.88%	48.25%	98.06%	98.06%	94.01%	93.62%	99.10%	77.71%	74.55%	95.67%
XS2-2	129934168	128221742	12822174200	48.30%	48.63%	97.62%	97.17%	94.01%	92.96%	98.68%	76.02%	72.88%	95.68%
XS2-3	129934168	128165600	12816560000	48.33%	48.70%	97.70%	97.26%	94.21%	93.24%	98.64%	75.14%	71.92%	95.66%
XS3-1	129383592	127630892	12763089200	48.60%	48.76%	98.01%	98.15%	93.85%	93.93%	98.65%	80.97%	77.75%	97.60%
XS3-2	129934168	127527340	12752734000	49.11%	49.25%	97.76%	97.01%	94.38%	92.68%	98.15%	78.27%	74.86%	97.13%
XS3-3	129934168	127919182	12791918200	48.73%	48.90%	97.85%	96.80%	94.53%	92.08%	98.45%	79.54%	76.13%	97.56%

Table S3. Primers used in this study.

Gene	Primer forward(F)	Primer reverse(R)	Use
PaF106G0700028583.01	TTGCTCGATTGACCCGTCTC	ACCAGACCAGGTAGTGTCCA	
PaF106G0700027669.01	CTCTGAGCTTCTGGGTGGTG	ACCACGAAGTTTCTAGGCCC	
PaF106G0700027524.01	GGATGTTACCCGGAACTTGG	CATACCTGTTTTGCCTAGTGGC	
PaF106G0700027260.01	ATGGGACGCTCTGGTCATTG	ACCGAGAGAACTTGAAGCCG	
PaF106G0700026687.01	ATGCAAGCATGGAACACCCT	CCAAGGAAACCCTGCCACAT	
PaF106G0600023388.01	TCGTTGCCTCCAATGGTGAT	TGTTCTTGGGTCTCAATAGCAGTG	
PaF106G0600023034.01	TCCTTCCAAAGCCCAGAGCA	GTGCCGATGATGATCACGTC	
PaF106G0400017352.01	TTTACTACAGAAAGCGGCAGC	TTTTGGGAAGTGGGTTTTGG	
PaF106G0300012468.01	GAACAGAGCAGGGTCAAGGAG	CTTGGCATATTCAGCACAGACC	
PaF106G0300012356.01	TATGCGATTCCTGCTGGGAC	TCGAAACCAGCCCATCCTTC	
PaF106G0300010939.01	GAAGAAGCAGGCTAGAAGGCAA	CCTTGTTGGATGAACTCGTGG	qPCR
PaF106G0200010557.01	GGAAGCACTGTTTCACTGTCTATG	GACAACTTCTCAGCCACACGTAC	
PaF106G0100002403.01	GGCCCAATGTTGCTCCCTAT	CAGGGCTTGTGCATTTTGCT	
PaF106G0100002035.01	TACGAAACACTCCCAGATGCG	ACCATTCCTCGGCCTTCCAT	
MTCONS_00054352	ACCCATCATGTGCTGCCAAT	TCCCACATCAGATTTCTTGGGTA	
MTCONS_00033405	GAGCATTGAGGGATGTACGGA	AGAGAAGTAAGTGCTAGGCCG	
LTCONS_00065464	GGGTGCCATTTTATAGAGGGAAG	AGGAAAGCAAGCTGAAGTAGCC	
LTCONS_00056403	CTGAAGGATTGACAAGCCCG	AGTGAAGAGGGTTTTGGAGGC	
LTCONS_00051642	CCATCTCTTTTGGCACCAGTC	CATGCACCCTATGACACCTGAT	
LTCONS_00032302	AGCACAGACCTCACCACATCG	TGGACTTGGATGAACGGATTG	
ACT	ACATTGTTCTTAGTGGTGGGTC	AGATTCGTCATACTCTGCCTTT	
PaPSY	ATGTGCAAGGAGGAATCAAT	CCCCAGTTGAAAAAGTCAGC	promoter activity

pBI121_PaPSY	tgaccatgattacgccTTCAAT<br>GTGCAAGGAGGAATC	taagggactgaccacccCTCAGA<br>AGAAAGGCAAGACT	
X15-PaPDS	CCATTTAAGGGTGCTGGTAAAAC	CACTTAAAAGAGGACTCCTGCTA	Overexpression
pBWA_X15-PaPDS	acggcatggacgagctctacATGTCT<br>CAGTGGGCTTGTGTCTCTG	tgaagacagagctagttacaTTAAGTAAATTC<br>AGACATAGATGAGCATGCGTGTTC	
JTY-PaPDS	CCATTTAAGGGTGCTGGTAAAAC	CACTTAAAAGAGGACTCCTGCTA	
pBWA_JTY-PaPDS	acggcatggacgagctctacATGTCT<br>CAGTGGGCTTGTGTCTCTG	tgaagacagagctagttacaTTAAGTAAATT<br>CAGACATAGATGAGCATGCGTGTTC	
TRVII_PaPSY	tgagtaaggttaccgaattcAGCC<br>CAGAATCAAAGGCTTCA	ggacatgcccgggcctcgagCCA<br>CACTGGCCATCTACTAGC	VIGS
TRVII_PaPDS	tgagtaaggttaccgaattcTCCCGA<br>ATACAGAAAATTGAGCTA	ggacatgcccgggcctcgagACAT<br>GTTACAGACATGTCAGCA	
